# The Influence of Menstrual Cycle and Androstadienone on Female Stress Reactions: An fMRI Study

**DOI:** 10.3389/fnhum.2016.00044

**Published:** 2016-02-16

**Authors:** Ka Chun Chung, Felix Peisen, Lydia Kogler, Sina Radke, Bruce Turetsky, Jessica Freiherr, Birgit Derntl

**Affiliations:** ^1^Department of Psychiatry, Psychotherapy and Psychosomatics, Medical Faculty, RWTH Aachen UniversityAachen, Germany; ^2^Jülich Aachen Research Alliance – Translational Brain MedicineAachen, Germany; ^3^Department of Psychiatry and Psychotherapy, Medical Faculty, University of TübingenTübingen, Germany; ^4^Neuropsychiatry Division, Department of Psychiatry, University of PennsylvaniaPhiladelphia, PA USA; ^5^Diagnostic and Interventional Neuroradiology, Medical Faculty, RWTH Aachen UniversityAachen, Germany; ^6^Institute for Neuroscience and Medicine, INM-1, Research Center JülichJülich, Germany

**Keywords:** menstrual cycle, stress, social threat, cortisol, hippocampus, amygdala

## Abstract

Communicating threats and stress via biological signaling is common in animals. In humans, androstadienone (ANDR), a synthetic male steroid, is a socially relevant chemosignal exhibited to increase positive mood and cortisol levels specifically in (periovulatory) females in positively arousing contexts. In a negative context, we expected that such effects of ANDR could amplify social evaluative threat depending on the stress sensitivity, which differs between menstrual cycle phases. Therefore, this fMRI study aimed to examine psychosocial stress reactions on behavioral, hormonal and neural levels in 31 naturally cycling females, between 15 early follicular (EF) and 16 mid-luteal (ML) females tested with ANDR and placebo treatment in a repeated-measures design. Regardless of odor stimulation, psychosocial stress (i.e., mental arithmetic task with social evaluative threat) led to elevated negative mood and anxiety in all females. A negative association of social threat related amygdala activation and competence ratings appeared in ML-females, indicating enhanced threat processing by ANDR, particularly in ML-females who felt less competent early in the stress experience. Further, ML-females showed reduced performance and stronger stress-related hippocampus activation compared to EF-females under ANDR. Hippocampal activation in ML-females also correlated positively with post-stress subjective stress. Contrarily, such patterns were not observed in EF-females or under placebo in either group. Strikingly, unlike passive emotional processing, ANDR in a stressful context decreased cortisol concentration in all females. This points to a more complex interaction of ovarian/gonadal hormones in social threat processing and stress reactivity. Our findings suggest that ANDR enhanced initial evaluation of self-related social threat in ML-females. Female stress reactions are related to stress sensitivity through enhanced awareness and processing of social cues in a stressful context, with menstrual cycle phase being a critical factor.

## Introduction

The presence of social chemosignals in nature facilitates social communication and also modulates emotional behaviors between conspecifics. An isolated compound in most human studies, 4, 16-androstadien-3-one (Androstadienone, ANDR), is secreted in male sweat, saliva or semen (see Krajnik et al., 2014 for a review). Females perceived ANDR (6.25 mM/pure crystal) differently than non-social odors and showed a range of enhanced physiological reactions, e.g., increased skin conductance, skin temperature (Bensafi et al., 2004; Wyart et al., 2007). After sniffing pure ANDR, females showed elevated cortisol and reduced negative mood ratings during a positively arousing context (Wyart et al., 2007), probably triggered by a stronger hypothalamic activation (Savic et al., 2001; Burke et al., 2012). Based on these earlier findings, it has been speculated that the properties of ANDR enhance autonomic arousal and probably act to increase sensitivity to social evaluative threat during psychosocial stress (Lübke and Pause, 2015).

Social evaluation and the gender of evaluators are contextual factors that reliably modulate psychosocial stress. Male evaluators increased cortisol levels in females during their follicular phase in the Trier Social Stress Test (TSST), while female evaluators increased cortisol levels in males (Duchesne et al., 2012). ANDR has been shown to discern masculine gender information (e.g., Zhou et al., 2014) and enhance attention on emotional information (Hummer and McClintock, 2009). Therefore, one might expect that presence of a male steroid should amplify female stress reactions resembling social evaluation by male experimenters. Prudently, in a negative context, ANDR may potentiate evaluation of self-related social threat (i.e., failing to perform under expectation), which may intensify psychosocial stress. In turn, this effect may be associated with the menstrual cycle.

Indeed, previous findings informing the current study suggested that stress reactions might be influenced by circulating ovarian hormones. More specifically, circulating ovarian hormones in different menstrual cycle phase could modulate social threat processing: In facial emotion processing tasks, evaluation of social threat was enhanced in females with higher progesterone concentration (Conway et al., 2007) and in females during their luteal phase (Derntl et al., 2008a; Maner and Miller, 2014). Similarly, exogenous progesterone administration in females enhanced social threat processing (van Wingen et al., 2008), suggesting a progesterone-driven effect. Typically, early-follicular females have lower progesterone and estradiol levels, while mid-luteal females exhibit higher ovarian hormones. Unlike high estradiol levels at periovulation (2nd and 3rd week of the menstrual cycle), high progesterone levels in the mid-luteal phase are always accompanied by a second peak of estradiol. This so-called progesterone-estradiol opposing effect appeared to exaggerate psychosocial stress reactions (Protopopescu et al., 2005; Andreano and Cahill, 2010). Therefore, the current study tested the possibility of increased stress reactions in females during the mid-luteal phase compared to females during the early follicular phase.

For stress-related cortisol reactivity cycle phase dependent effects have been shown previously, indicating higher responsivity in mid-luteal females compared to females during the follicular phase (Kirschbaum et al., 1999). This suggests that ovarian hormone levels associated with different menstrual cycle phases have differential effect on cortisol reactivity following psychosocial stress. More recently, Duchesne and Pruessner (2013) investigated behavioral and hormonal effects of menstrual cycle phase on the stress response and observed no female group differences in cortisol concentration in using the TSST. Interestingly, significant correlations between negative affect and peak cortisol emerged albeit with contrasting group results. Maki et al. (2015) demonstrated that the stress effect of the TSST on emotional retrieval was further related to individual differences in cortisol response in females during the follicular but not luteal phase (see also Het et al., 2005). This further supports the notion that cycle phase could modulate social threat processing and cortisol reactivity. Since most previous stress studies employed a mixed gender or male-only sample, there is an increasing demand for exclusively female samples investigated across menstrual cycle phases in stress studies (Cahill, 2006).

Regarding the neural basis of psychosocial stress, previous work points toward an involvement of the amygdala (detection of social threat), hippocampus (encoding of social threat), anterior cingulate cortex (emotional processing), and orbitofrontal cortex (regulation of emotional responses) (e.g., Goldstein et al., 2005; Pruessner et al., 2008). Preceding neuroimaging studies using the Montreal Imaging Stress Task (MIST), which combines mental arithmetic and social evaluative threat, have reliably induced psychosocial stress in a neuroimaging environment (Dedovic et al., 2005; Pruessner et al., 2008). Recently, Albert et al. (2015) showed stronger hippocampal activation in females at periovulation compared to early follicular females. Thus, these findings are a first hint that menstrual cycle not only modulates cortisol reactivity but also neural activation during psychosocial stress.

Translating these stress-related processes to the current study, it is worth noting that unlike passive emotional processing, psychosocial stress in the MIST incorporated elements of self-related threat, motivation, and self-esteem (Albert et al., 2015) to probe real-life stressful situations. Anticipatory cognitive appraisal and self-esteem were shown to predict subsequent psychosocial stress reactions (Gaab et al., 2005; Pruessner et al., 2010; Juster et al., 2012). Moreover, within the stress coping literature, individuals with more psychosocial resources, such as higher self-esteem, may have higher thresholds for social threat (Taylor et al., 2008; Pruessner et al., 2010). Compared to those, individuals with lower self-esteem may feel less competent in confrontation of social threat and they may be characterized by higher sensitivity to social threat signaled by heightened amygdala activity. In keeping with the proposition of enhanced social threat processing in mid-luteal females, the initial impact of ANDR on their psychosocial stress reaction to such threat might be stronger.

Taken together, chemosensory signals influence psychosocial stress reactions in humans, akin to a variety of other species (Albrecht et al., 2010; Lübke et al., 2014). Moreover, females were shown to be more sensitive to male odor cues than males (e.g., Zhou and Chen, 2009). Therefore, we investigated whether a socially relevant chemosignal, ANDR, combined with social evaluative threat (within a psychosocial stress task, MIST) modulates the behavioral, hormonal and neural stress response differently in early follicular vs. mid-luteal females in a repeated-measures, placebo-treatment fMRI experiment.

Based on previous findings (Maner and Miller, 2014), we hypothesized that mid-luteal females compared to early follicular females would show higher sensitivity to social threat, indicated by stronger mood changes (e.g., feeling of being competent) and reduced performance, as well as stronger hippocampal activation during stress. Moreover, we hypothesized that this heightened sensitivity and accompanying neural activation would be enhanced under ANDR. Regarding cortisol, we expected higher cortisol concentrations under ANDR in all females (Wyart et al., 2007), irrespective of cycle group (cf. Duchesne and Pruessner, 2013). Following results from Taylor et al. (2008) and Pruessner et al. (2010), we hypothesized that amygdala activation in the ANDR stress condition would be negatively associated with self-esteem (Taylor et al., 2008) in mid-luteal females who were typically more aware of self-related threat (Maner and Miller, 2014).

## Materials and Methods

### Ethics Statement

The local ethics committee at the Medical Faculty of RWTH Aachen University approved the current study. The experimental protocol was carried out in accordance with the provisions of the World Medical Association Declaration of Helsinki.

### Sample

Thirty-one healthy, right-handed, heterosexual females, aged 19–38 years (*M* = 26.94, *SD* = 4.5) were recruited. Only naturally cycling females who reported regular menstrual cycle length (26–30 days) for the last 6 months were included. In the screening interview, at least three previous cycle lengths were surveyed (except for two subjects with only one previous cycle surveyed). We relied on extensive self-report measures for menstrual cycle status. Participants were asked to call as soon as their menstrual period started (day 1) and were distributed to one of two experimental groups: early follicular (EF; day 2–7 after onset of menses; *n* = 15) or mid-luteal (ML; day 20–25 after onset of menses; *n* = 16) (see Supplementary Table S1 for details of testing day (day within the actual cycle) and days until next menstruation as well as general length of menstruation for all mid-luteal women). To ensure that all testing sessions were conducted in the correct time window, all females reported the beginning of their next menstruation. No group difference in age, years of education, body-mass-index, cycle length, or chronic stress (Trier Inventory for the Assessment of Chronic Stress; Schulz and Schlotz, 1999) emerged (all *t*s ≤ 1.418, *p*s ≥ 0.126; see **Table 1**).

**Table 1:** Sample description of females in early follicular (EF) and mid-luteal (ML) group representing means (standard deviation), *t*- and *p*-values.

	EF (*n* = 15)		ML (*n* = 16)		*t*	*p*-value
Cycle day (post-menstruation)	4.87	(1.0)	22.40	(1.5)	–37.80	<0.001
Cycle length	28.07	(1.9)	28.75	(1.3)	0.55	0.59
Between testing (days)	40.40	(51.1)	50.25	(49.3)	–0.55	0.59
Age	26.40	(3.9)	27.44	(5.1)	–0.64	0.53
Years of education	16.13	(2.4)	15.00	(1.7)	1.41	0.17
BMI	23.11	(3.4)	22.71	(7.4)	0.19	0.85
BDI	5.00	(6.9)	4.13	(4.2)	0.43	0.67
TMT-A (sec)	16.08	(5.1)	16.63	(4.7)	–0.29	0.78
TMT-B (sec)	28.97	(10.4)	29.37	(6.4)	–0.12	0.91
Verbal intelligence^a^	33.73	(2.8)	33.20	(3.3)	0.48	0.63
Trait anxiety^a^	31.00	(11.8)	33.19	(6.1)	–0.66	0.52
MONEX-40^a^	32.73	(3.4)	32.94	(3.0)	–0.18	0.86

*Body-mass-index (BMI), verbal intelligence (raw scores; Wortschatztest, WST; Schmidt and Metzler, 1992), visual attention and task switching (Trail–Making Test, TMT; Reitan, 1956), depression scale (BDI-II; Hautzinger et al., 2006), trait anxiety (STAI; Laux et al., 1981), 40-item Monell Extended Sniffin’ Sticks Identification Test (MONEX-40, Freiherr et al., 2012).*^a^*Data represents in raw score.*

Exclusion criteria included the use of oral contraceptives and other hormonal and psychotropic medication or illicit drug use in the last 6 months as well as prior or present neurological and psychological illness (confirmed via a standardized clinical interview SCID; Wittchen et al., 1997). None of the females were pregnant or breast feeding within the last 12 months. Due to the nasal delivery method of the steroid, those who frequently or within the previous 3 days had nosebleeds, chronic nasal disease, rhinitis, upper respiratory tract infections or lung disease were excluded. Subjects smoking up to five cigarettes/day except on the day of testing were included.

### fMRI Stress Paradigm

**FIGURE 1:**
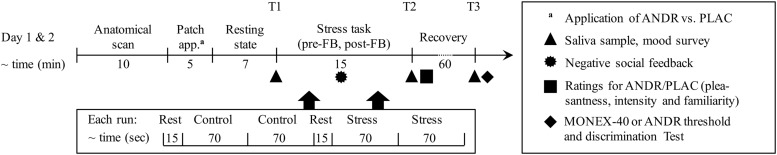
**Timeline of the paradigm of each testing day: patch application order of androstadienone (ANDR) and placebo (PLAC) were randomized for all females.** To ensure ANDR to take effect, a 7 min resting state was performed before the stress task. There were two blocks of the stress paradigm, and experimenters gave negative feedback between the two blocks. Between each condition in each block, a 6–8 s jittered fixation cross was presented.

We applied the MIST, (Dedovic et al., 2005; Pruessner et al., 2008), a mental arithmetic task with social evaluative threat and uncontrollability elements (Dickerson and Kemeny, 2004). This sequence of three conditions (rest-control-rest-stress) in a block design was presented in two blocks (pre-feedback [pre-FB] and post-feedback [post-FB], see **Figure 1** for the order of conditions). In the rest condition, a black fixation cross was presented. In the control condition, participants were instructed to solve the arithmetic problems and respond as quickly as possible in a rotary-dial interface with a three-button touch pad. In the stress condition, social evaluative threat and sense of uncontrollability were increased by variable time pressure and (mock) performance monitoring on a visual scale (in green, yellow, and red, the latter indicating low performance), which was of particular interest for the effect of ANDR on social evaluative threat. Task speed and difficulty were adaptive with overall 40–50% accuracy with the majority of errors committed in the stress condition (see Dedovic et al., 2005). Pressure and motivation to perform were assessed via the number of total problems, while the effect of stress on performance was assessed by the number of correct responses. For negative social feedback, the male investigator verbally discredited subjects’ poor performance via microphone. Participants were asked to improve their performance in the post-FB block. This task sequence was the same for ANDR and placebo (PLAC) treatment testing (see **Figure 1**).

### Preparation of ANDR and PLAC

A 250 μM ANDR (with a purity of ≥99%; Steraloids Inc, Newport, RI, USA; diluted in propylene glycol) solution was masked with 1% musk oil (Sigma–Aldrich, Deisendorf, Germany). This concentration was chosen since it was previously determined as an absolute detection threshold (Lundström et al., 2003) and shown to increase autonomic arousal in females (Jacob et al., 2001). PLAC was solely a 1% musk oil solution (in propylene glycol). ANDR or PLAC was pipetted on a cotton pad with one-direction permeability and attached on the upper lip to avoid transdermal exposure (Albrecht et al., 2010).

### Procedure

Each woman performed two sessions (ANDR vs. PLAC) on two different testing days (within-subject), and the treatment sequence was randomized with approximately 4 weeks gap. Due to diurnal variation of cortisol concentration, all participants were measured in the afternoon between 2 and 5 pm, with both sessions starting at the same time. ANDR or PLAC was applied prior the resting-state scan that was performed to avoid confounding stressors (i.e., MRI scanner environment) and to ensure that ANDR took effect (after approximately six min; Jacob and McClintock, 2000). After 7 min (resting-state scan, which data will be reported elsewhere), the first block of the stress task (pre-FB) began and was followed by negative verbal feedback. Then the second block (post-FB) followed. Both blocks lasted for about 7 min each (see **Figure 1**).

Immediately following the MRI session (T2; see **Figure 1**), participants rated the odor of ANDR and PLAC with regards to pleasantness, intensity and familiarity on 10 cm visual analog scales (VASs). Furthermore, threshold tests for ANDR were performed after the last cortisol sample (T3) was collected on the testing day using ANDR (see **Figure 1**) in order to determine each participant’s discrimination ability (ANDR versus PLAC) and their sensitivity to ANDR (see Supplementary Materials). Additionally, general ability to identify odors using the 40-item Monell Extended Sniffin’ Sticks Identification Test (MONEX-40, Freiherr et al., 2012) was assessed on the testing day using PLAC.

### Stress Assessment and Saliva Samples

To control for confounding cortisol changes, participants were asked to refrain from exercise and alcohol the day before testing; from black tea, caffeine, highly sugared, and carbonated beverages on the testing day; and from eating and drinking, except water, 2 h prior to testing. Before entering the MRI scanner, participants completed the State-Trait Anxiety Inventory (STAI; Laux et al., 1981). After the MRI measurement, state anxiety was surveyed again. To assess stress levels, subjective mood (Positive and Negative Affect Scale [PANAS]; Watson et al., 1988), subjective stress and competence ratings were verbally surveyed (“*How stressed/competent do you feel at the moment*”) on a five-point Likert scale, since individuals with low self-esteem seem to be particularly sensitive to psychosocial stress (Taylor et al., 2008; Juster et al., 2015a). These subjective mood ratings and salivary cortisol samples were drawn at three time points: 10 min after exposure to ANDR or PLAC, i.e., immediately before the stress task (T1), 15 min (T2), and 60 min (T3) after the stress experience (see **Figure 1**). Saliva samples were analyzed by the local laboratory (Uniklinikum Aachen, Germany) using an ElectroChemiLumineszenzImmunoAssay (ECLIA; functional sensitivity <8.5 nml/l). Data for skin conductance response (SCR) was collected during MRI scanning and analyzed (see Supplementary Materials for information on acquisition parameters and statistical analysis and results).

### Statistical Analysis

Statistical analysis was conducted using IBM SPSS statistics for Windows, Version 20.0 (IBM Corp., Armonk, NY, USA). Independent sample *t*-tests were applied to test for group differences in sample characteristics. Subjective and psychophysiological variables, cortisol levels and SCR data were tested for normal distribution using Kolmogorov–Smirnov tests; logarithmic transformation (*y* = log10[x+1]; Boucsein et al., 2012) was applied on cortisol data since assumption of normal distribution was violated.

Significance level was set to alpha <0.05. Estimates of effect size (partial-eta squared) will be reported for significant effects. For significant interactions, all pairwise comparisons will be Bonferroni-corrected. Greenhouse–Geisser correction was applied to the degrees of freedom of the repeated factors when requirements were not met.

#### Subjective Mood

Subjective stress and PANAS ratings were analyzed by a 2 × 3 × 2 repeated measures ANOVA (rmANOVA) with the factors treatment (ANDR/PLAC), time (T1/T2/T3) and group (EF/ML). For state anxiety and competence ratings, we performed a 2 × 2 × 2 rmANOVA, as only two time points were assessed (before vs. after stress induction).

#### Task Performance

The number of total problems solved and correct responses were analyzed by a 2 × 2 × 2 × 2 rmANOVA with the factors treatment (ANDR/PLAC), feedback (pre-FB/post-FB), condition (control/stress), and group (EF/ML).

##### Cortisol

Log-transformed data regarding salivary cortisol levels were analyzed using a 2 × 3 × 2 rmANOVA with the factors treatment (ANDR/PLAC), time (T1/T2/T3), and group (EF/ML).

*Correlation analyses* were applied to investigate the relationship between stress ratings, cortisol levels, subjective mood and stress task performance. For variables that did not survive the Kolmogorov–Smirnov normality test, Spearman’s correlations rather than Pearson’s were carried out separately for both groups (EF/ML) and treatment (ANDR/PLAC): subjective stress, competence ratings (T1, T2, and T2-T1) with cortisol (T1, T2, T2-T1) and performance (overall total correct responses in pre- and post-FB block). To test for significant group differences of these significant correlations, the Fisher’s Z-transformation was applied to the correlation coefficients.

### MRI Data Acquisition, Preprocessing and Analyses

Functional and anatomical imaging data were acquired with a 3T TIM Trio scanner (Siemens Medical Solutions, Erlangen, Germany) equipped with the manufacturer’s standard 12-channel head coil. First, a high-resolution anatomical image was acquired using an MPRAGE (3-D Magnetization Prepared Rapid Gradient Echo) sequence consisting of 160 sagittal slices (TR = 1900 ms, TE = 2.52 ms, TI = 900 ms, 1 × 1 × 1 mm resolution, field of view (FOV) 250 × 250 mm, slice oversampling = 18.2%, flip angle [FA] = 9°). For the task, 34 ascending slices were acquired with a gradient-echo EPI-sequence with distortion correction (TR = 2000 ms, TE = 28ms, 3.3 × 3.3 × 3.3 mm resolution, FOV = 210 × 210 mm, matrix size = 256 × 256, and FA = 77°).

Functional imaging data processing was performed with the Statistical Parametric Mapping software (SPM8; Wellcome Department of Imaging Neuroscience, London, UK) implemented in Matlab (Mathworks Inc., Sherborn, MA, USA) using standard settings unless specified differently. This included realignment, coregistration to T1-image, spatial normalization to MNI (Montreal Neurological Institute) space (Ashburner and Friston, 2005), and spatial smoothing (8 mm Gaussian kernel). Pre-processed images were then analyzed using a general linear model for each subject.

At the first level, main effects for each participant were computed by applying appropriate contrasts for each condition (pre-FB rest, pre-FB control, pre-FB stress, post-FB rest, post-FB control, post-FB stress). Three translation and three rotation estimates generated by the realignment step served as nuisance regressors for head motion correction. The resulting con-images were further fed into a second-level group analysis. Data from three participants were discarded due to movement artifacts (>3 mm). The remaining 28 females (13 EF-females; 15 ML-females) were included in the analyses of the functional data. For all other analyses we relied on the full sample.

The hippocampus and amygdala were selected based on the menstrual cycle-related stress circuitry reported in previous studies (Goldstein et al., 2005; Andreano and Cahill, 2010; Albert et al., 2015). Anatomical regions of interest (ROIs) for the bilateral hippocampus and amygdala were based on predefined regions in the AAL atlas (Tzourio-Mazoyer et al., 2002). All masks of the ROIs were extracted by SPM Marsbar toolbox (Brett et al., 2002). Mean activations for each condition in each participant for both hippocampus and amygdala were exported and analyzed in SPSS. For the amygdala and hippocampus, 2 × 2 × 2 × 2 × 2 rmANOVAs with laterality (left/right), treatment (ANDR/PLAC), feedback (pre-FB/post-FB), condition (control/stress) and group (EF/ML) were conducted.

Previous studies reported self-esteem as a major factor affecting neural stress responses (Taylor et al., 2008); therefore, Spearman’s correlations between subjective stress, competence and neural activations in the pre- and post-FB blocks were applied, whereas Pearson’s correlations were applied between cortisol levels and negative mood ratings (T1, T2, and T2-T1), performance, and neural activations in the pre- and post-FB blocks (parameter estimates as derived from ROI analysis). This tested for brain-behavior associations separately for both groups and treatment. To test for significant group differences of these significant correlations, the Fisher’s Z-transformation was applied to the correlation coefficients to formally test for significant differences.

## Results

### Subjective Ratings and Mood

The rmANOVAs revealed a significant time effect (all *F*s ≥6.694, *p*s ≤ 0.003, $\eta_{p}^{2}\geq 0.188$), indicating increased post-stress ratings of subjective stress, state anxiety, and negative mood. Disentangling the significant time effect revealed significantly higher subjective stress ratings (T2 > T1 & T3; all *p*s < 0.001) and higher negative mood (T2 > T1; *p* = 0.003) after the stress induction. State anxiety was significantly higher after compared to before the MRI session (*p* = 0.003). For competence ratings, a significant main effect for time (*F*_1,29_ = 16.72, *p* < 0.001, $\eta_{p}^{2}=0.379$) appeared: Participants reported feeling less competent after compared to before stress experience (T2 < T1, *p* = 0.001). Also, a treatment-by-group interaction was observed (*F*_1,29_ = 5.09, *p* = 0.032, $\eta_{p}^{2}=0.149$), with ML-females rating themselves as less competent on ANDR compared to PLAC (*p* = 0.058). EF-females did not report such difference (*p* = 0.830) (see **Table 2**). No group difference emerged (*p* = 0.277). All other effects and interactions were not significant (all *p*s ≥ 0.386) (see **Table 2** for means).

**Table 2:** Subjective stress, negative mood, and competence ratings and raw values of cortisol concentration (μg/dl), represent mean (standard deviation) for androstadienone (ANDR) and placebo (PLAC) treatment in early follicular (EF) and mid-luteal (ML) group.

	EF (*n* = 15)		ML (*n* = 16)	
	ANDR	PLAC	ANDR	PLAC
**Stress**				
T1	1.76 (1.1)	1.60 (0.8)	1.72 (0.7)	1.62 (0.7)
T2	2.80 (1.4)	3.07 (1.5)	2.56 (1.0)	2.60 (0.8)
T3	1.53 (1.1)	1.60 (0.7)	1.78 (0.8)	1.94 (0.9)
**Negative mood**				
T1	11.67 (3.0)	12.40 (3.6)	13.44 (4.9)	13.25 (3.3)
T2	13.27 (4.6)	12.80 (4.0)	15.00 (3.6)	13.81 (3.4)
T3	11.56 (2.0)	11.80 (2.4)	11.96 (1.9)	11.75 (1.4)
**Competence**				
T1	2.50 (0.9)	2.27 (1.2)	2.47 (1.0)	2.81 (0.8)
T2	2.00 (1.2)	1.67 (0.9)	2.19 (1.2)	2.38 (1.0)
**Cortisol**				
T1	0.34 (0.1)	0.39 (0.1)	0.40 (0.2)	0.49 (0.3)
T2	0.30 (0.1)	0.35 (0.1)	0.38 (0.2)	0.47 (0.2)
T3	0.28 (0.1)	0.32 (0.1)	0.36 (0.2)	0.37 (0.2)

*T1 (immediately before the stress task), (T2) 15 min, and (T3) 60 min after the stress experience.*

### Task Performance (Total Responses, Correct Responses)

**FIGURE 2:**
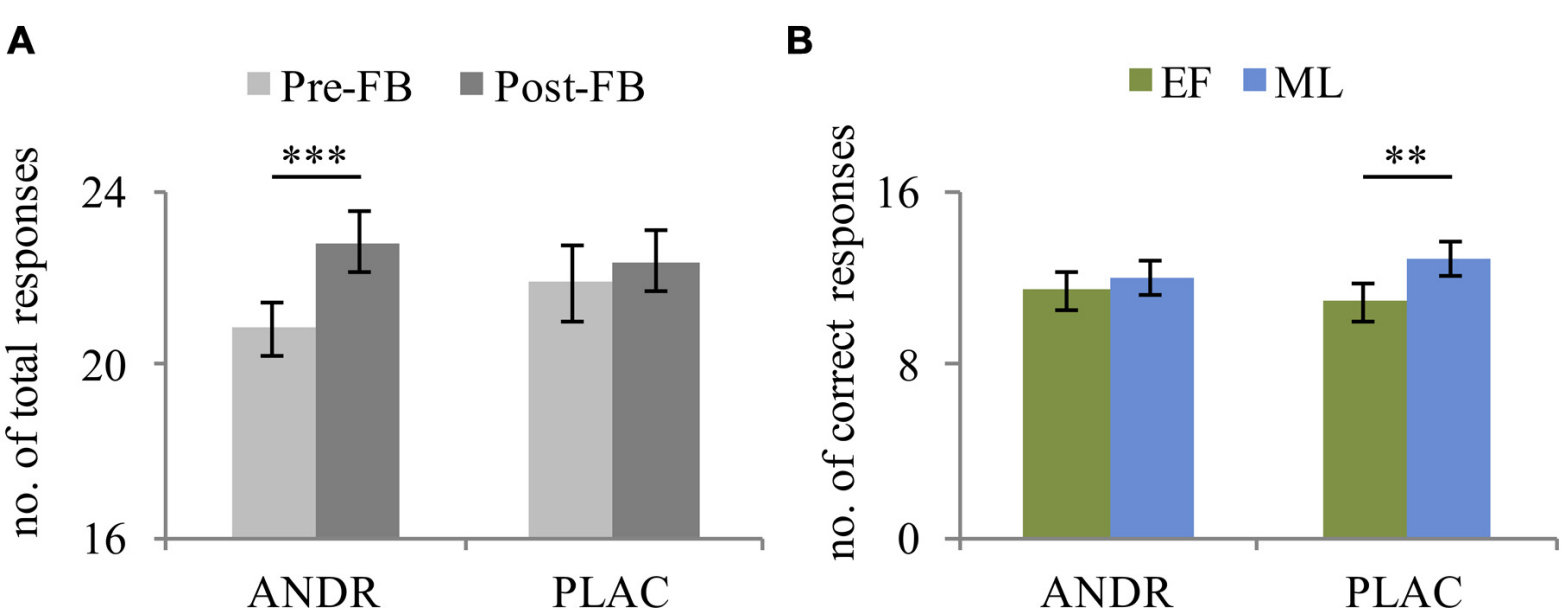
**Mean and standard error of **(A)** total responses of all participants (*n* = 31) illustrating the treatment-by-feedback interaction and **(B)** correct performance of early follicular (EF, *n* = 15) and mid-luteal (ML; *n* = 16) group depicting the treatment-by-group interaction.** ^∗∗^Significant difference at *p* ≤ 0.01; ^∗∗∗^Significant difference at *p* ≤ 0.001.

The rmANOVA revealed a main effect of condition in total responses (*F*_1,29_ = 507.23, *p* < 0.001, $\eta_{p}^{2}=0.946$), indicating more processed problems in the stress compared to the control condition. Also, a significant effect of feedback was observed (*F*_1,29_ = 9.522, *p* = 0.004, $\eta_{p}^{2}=0.247$), as participants solved fewer problems before negative feedback compared to after. Moreover, a significant treatment-by-feedback interaction was evident (*F*_1,29_ = 4.61, *p* = 0.040, $\eta_{p}^{2}=0.137$): before negative feedback was given, a trend toward fewer problems being solved on ANDR compared to PLAC occurred (*p* = 0.053). Additionally, during ANDR application, fewer problems were solved before compared to after negative feedback (*p* = 0.001) (see **Figure 2A**). No significant group effect emerged (*p* = 0.146).

For correct responses, the rmANOVA revealed a significant main effect of condition (*F*_1,29_ = 35.71, *p* < 0.001, $\eta_{p}^{2}=0.552$), with more correct responses during the stress than control condition. Again, a trend toward a significant feedback effect emerged (*F*_1,29_ = 4.13, *p* = 0.051, $\eta_{p}^{2}=0.125$), indicating fewer correct responses before feedback than after. Importantly, a significant treatment-by-group interaction was observed for correct responses (*F*_1,29_ = 9.73, *p* = 0.004, $\eta_{p}^{2}=0.251$), indicating that the ML-females made fewer correct responses on ANDR than on PLAC (*p* = 0.011), which was not the case for the EF-females (*p* = 0.098). Thus, the performance level of ML-females decreased under ANDR (see **Figure 2B**). No significant group effect emerged (*p* = 0.291).

### Cortisol Levels

The rmANOVA revealed no group effect (*F*_1,29_ = 2.132, *p* = 0.155), but a significant treatment effect (*F*_1,29_ = 5.47, *p* = 0.026, $\eta_{p}^{2}=0.159$) with lower cortisol levels on ANDR and a significant time effect (*F*_1,28_ = 13.78, *p* < 0.001, $\eta_{p}^{2}=0.322$). *Post hoc* tests disentangling the time effect showed a higher concentration of cortisol at T1 and T2 compared to T3 (*p* < 0.001; *p* = 0.015, respectively), while no significant difference emerged between T1 and T2 (*p* = 0.073) (see **Table 2** for means).

### Correlation Analyses for Behavioral Parameters

During PLAC treatment, competence ratings at T2 negatively correlated with stress ratings at T2 (Spearman’s *r* = –0.68, *p* = 0.005) for EF-females, which was not observed at T1 or T2 in ML-females (all *p*s ≥ 0.513) or under ANDR (all *p*s ≥ 0.072). Neither stress nor competence ratings correlated with cortisol levels (all *p*s ≥ 0.152) or performance (all *p*s ≥ 0.086) in either group for both treatments.

### Region-of-Interest Analyses

#### Amygdala

**FIGURE 3:**
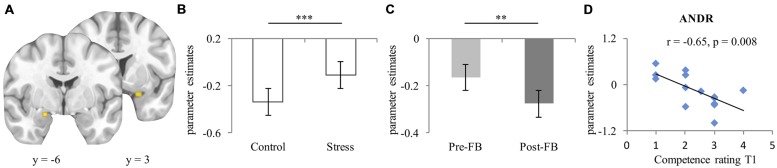
**(A)** Peak activation coordinate of amygdala left (–19, –6, –17), right (24, 3, –21); **(B)** Mean parameter estimates extracted from the peak voxel of left and right amygdala depicting the main effect of condition, and **(C)** Main effect of feedback; **(D)** Scatterplot of the correlation of activation of right amygdala during stress experience in the pre-FB block and competence rating at T1 in ML-females treated with ANDR. Data represents mean and standard error. ^∗∗^Significant difference at *p* ≤ 0.01; ^∗∗∗^Significant difference at *p* ≤ 0.001.

For bilateral amygdala (left [–19, –6, –17] k = 22, right [24, 3, –21] k = 31 [see **Figure 3A**]), a significant condition effect (*F*_1,26_ = 13.57, *p* = 0.001, $\eta_{p}^{2}=0.343$) emerged, indicating more activation in the stress compared to the control condition (see **Figure 3B**). No group difference (*p* = 0.453) appeared. Moreover, a main effect of feedback appeared (*F*_1,26_ = 7.17, *p* = 0.013, $\eta_{p}^{2}=0.216$), with more activation in the pre-FB block compared to the post-FB (see **Figure 3C**). A significant laterality-by-condition-by-group interaction (*F*_1,26_ = 4.668, *p* = 0.004, $\eta_{p}^{2}=0.152$) also emerged. *Post hoc* analyses provide evidence that EF-females showed less activation of the left amygdala than the right in the control condition (*p* = 0.016) but not in the stress condition (*p* = 0.403). However, this interaction was not observed in ML-females (*p* = 0.589). All other main effects or interactions remained non-significant (all *p*s ≥ 0.062).

#### Hippocampus

**FIGURE 4:**
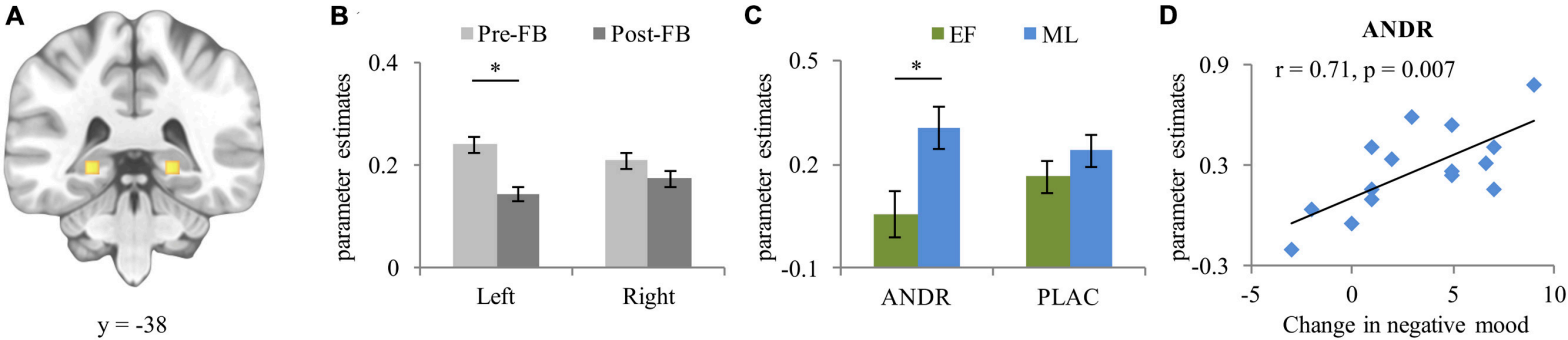
**(A)** Peak activation coordinate of left (–22, –38, –4) and right (21, –38, –4) hippocampus; **(B)** Mean parameter estimates extracted from the peak voxel of left and right hippocampus depicting the laterality-by-feedback interaction, and **(C)** Treatment-by-group interaction; **(D)** Positive correlation between increase in negative mood (T2-T1) and activation of the hippocampus during post-FB stress experience in ML-females treated with ANDR. Data represents mean and standard error. ^∗^Significant difference at *p* ≤ 0.05.

For bilateral hippocampus (left [–22, –30, –4] k = 128, right [21, –30, –4] k = 158 [see **Figure 4A**]), a significant condition effect (*F*_1,26_ = 21.97, *p* < 0.001, $\eta_{p}^{2}=0.458$) occurred, with stronger activation in the stress than control condition. A significant group effect emerged (*F*_1,26_ = 5.67, *p* = 0.025, $\eta_{p}^{2}=0.179$), with ML-females showing stronger hippocampus activation than EF-females. Moreover, a significant laterality-by-feedback interaction (*F*_1,26_ = 7.33, *p* = 0.012, $\eta_{p}^{2}=0.220$) emerged. *Post hoc* analysis of the significant laterality-by-feedback interaction indicated stronger left hippocampus activation in the pre-FB than in the post-FB block (*p* = 0.024), but not in the right hippocampus (*p* = 0.363) (see **Figure 4B**). A marginally significant treatment-by-group interaction (*F*_1,26_ = 3.54, *p* = 0.058, $\eta_{p}^{2}=0.116$) emerged. Disentangling this interaction revealed that on ANDR, ML-females showed stronger activation than EF-females (*p* = 0.021) (see **Figure 4C**), while this difference did not occur during PLAC (*p* = 0.258). A laterality-by-feedback-by-condition-by-group interaction (*F*_1,26_ = 4.232, *p* = 0.050, $\eta_{p}^{2}=0.139$) and a laterality-by-treatment-by-feedback-by-condition-by-group (*F*_1,26_ = 5.539, *p* = 0.026, $\eta_{p}^{2}=0.176$) interaction emerged, however, *post hoc* ANOVAs did not generate significant lower order interactions (all *p*s ≥ 0.083). No other main effect reached significance (all *p*s ≥ 0.093).

### Correlation Analyses for Neural Activation

#### Amygdala

During ANDR treatment mean bilateral amygdala activation in the pre-FB block correlated negatively with competence ratings at T1 in ML-females (Spearman’s rho *r* = –0.65, *p* = 0.008, see **Figure 3D**), but not in EF-females (Spearman’s rho *r* = 0.30, *p* = 0.319). This association was significantly different between groups (*Z* = –2.53, *p* = 0.006). No other correlations reached significance in ML-females (all *p*s ≥ 0.324). Moreover, no significant associations emerged in EF-females (all *p*s ≥ 0.103).

#### Hippocampus

During ANDR treatment mean bilateral hippocampal activation in the post-FB block correlated positively with change in negative mood (T2-T1) in ML-females (Pearson’s *r* = 0.71, *p* = 0.007, see **Figure 4D**), but not in EF-females (Pearson’s *r* = –0.42, *p* = 0.159). This association was significantly different between groups (*Z* = 3.12, *p* = 0.001). No other correlations reached significance in either ML-females (all *p*s ≥ 0.122) or EF-females (all *p*s ≥ 0.122).

### Odor Quality of ANDR vs. PLAC

No significant main effect of group, treatment or interactions emerged for ratings of odor pleasantness (all *p*s ≥ 0.433), intensity (all *p*s ≥ 0.525), or familiarity (all *p*s ≥ 0.359) (see **Table 3**). Threshold tests exhibited that two EF-females and two ML-females were anosmic to ANDR. Since it is still under debate whether conscious exposure to ANDR is necessary (Jacob and McClintock, 2000; Lundström et al., 2003) and exclusion of these participants did not change the direction of the reported results, we decided to include them in the final sample. In the additional discrimination tests, six females of each group could discriminate between ANDR and PLAC (see Supplementary Materials).

**Table 3:** Perception of ANDR and placebo odor (PLAC) in early follicular (EF) and mid-luteal (ML) group represent mean (standard deviation), *t*- and *p*-values.

		EF (*n* = 15)	ML (*n* = 16)	*t*	*p*-value
**Pleasantness**					
	ANDR	6.03 (3.0)	5.79 (1.6)	0.27	0.79
	PLAC	5.83 (2.0)	5.32 (1.5)	0.79	0.44
**Intensity**					
	ANDR	5.20 (3.0)	5.09 (1.9)	0.12	0.91
	PLAC	4.65 (2.3)	4.99 (2.0)	–0.45	0.66
**Familiarity**					
	ANDR	4.31 (2.9)	4.29 (3.1)	–0.04	0.97
	PLAC	4.75 (3.2)	4.87 (3.1)	0.22	0.83

## Discussion

The aims of the current study were twofold. First, we explored the impact of menstrual cycle phase on neural stress reactions in females at different stages of the menstrual cycle. Second, we examined the effects of ANDR on the female stress response during social threat with performance stressors, particularly prior to negative social evaluation (negative verbal feedback), that is, during the initial impact (pre-feedback) and reaction (post-feedback) to social threat.

While no group differences in performance appeared, ML-females showed enhanced activation of the hippocampus compared to EF-females, as hypothesized. Regarding effects of ANDR, overall cortisol levels were surprisingly lower in all females. All females showed increased performance after negative feedback. Moreover, behavioral and neural stress reactions in response to psychosocial stress and chemosensory signals differed depending on menstrual cycle phase: ANDR enhanced stress reactions in ML-females, indicated by fewer correct responses. Regarding neural activation, a general effect of stress was observed for all pre-defined regions, with stronger activation in the stress condition. We confirmed a negative association between stress activation of the amygdala and competence ratings during ANDR treatment. In line with our expectation, a positive association between post-stress subjective stress and hippocampus activation occurred in ML-females, but lacked in EF-females or under placebo odor exposure.

### ANDR Enhanced Self-Related Social Threat

We predicted that ANDR would enhance processing of self-related social threat. As previously shown, stressed females tended to be more cautious and take longer in decision-making (Lighthall et al., 2011). This stress effect appeared to be stronger under exposure to ANDR: ANDR exposure led to processing of fewer tasks during the pre-FB block of the MIST in all females, in which enhanced assessment of emerging social threat occurred. ANDR application has been shown to modulate attentional processes toward social cues, such as enhancing attention to emotional signals (Hummer and McClintock, 2009) and increasing perceived pain intensity (Villemure and Bushnell, 2007). The attention to self-related negative social cues might be augmented by the social saliency of ANDR. Therefore, we interpret the decreased performance level in the pre-FB block as a sign for elevated stress during the initial processing of self-related threat during the social evaluation (Dickerson and Kemeny, 2004).

Low self-esteem was shown to be one major modulator for self-related threat and social threat regulation (Pruessner et al., 2008; Taylor et al., 2008). Feeling less competent before the MIST could reflect a stress reaction to the anticipation of social evaluation. Competence ratings in ML-females exposed to ANDR decreased, as a result this might modulate subsequent behavioral and neural stress responses. Similar to sweat, ANDR enhanced biologically relevant information. ML-females could be more attuned to social evaluative threat. Previously, Dalton et al. (2012) indicated that men rated females as less competent and more stressed when the same female’s stress sweat was applied to the male rater in a video observation task. Our novel findings of the interaction between social chemosensory signals and menstrual cycle extend the previously observed complex social behavior outcome: ML-females in the current study were susceptible to negative bias as they attended to psychosocial stressors, this effect was stronger than in EF-females. Thus, ML-females may tend to perceive social evaluative threat as more self-related than the EF-females. Therefore, we suggest that influence of ANDR on stress reactions is based on the subjective assessments of stressful situations. While self-esteem, a characteristic trait, is related to subjective stress (Taylor et al., 2008; Juster et al., 2015a), feeling of being less competent can be viewed as a stress reaction of how overwhelming the task was at that moment.

### Female Cortisol Reactivity Under ANDR and Psychosocial Stress

Against our hypothesis, overall cortisol levels were lower during ANDR application in both cycle groups. Although we used a threshold used in previous studies that demonstrated a mood modulation effect (Hummer and McClintock, 2009), we were aware of the higher ANDR concentration used in a positively or sexually arousing context in previous studies (Bensafi et al., 2004; Wyart et al., 2007). Nevertheless, the lack of cortisol increase under ANDR may not be contradictory to previous findings. Social interaction was limited in all previous ANDR studies; male experimenters in our test sessions were a social stressor rather than a possible source of positive arousal (Wyart et al., 2007). Thus, the influence of ANDR on cortisol levels might arise due to social interactions demanded by different paradigms. Considering that participants should have enhanced attention on the social threat in the stressful context with increased negative mood on both treatments here, a positive effect on mood and arousal for females under ANDR is deemed less likely.

In the current study, ANDR appeared to modulate the hypothalamic-pituitary-adrenal (HPA axis) response selectively (see also later discussion of hippocampus activation relating to cortisol). Het et al. (2012) reported that lower cortisol levels were related to higher negative mood after the stress experience, suggesting a reinstatement of homeostasis (see also Young and Altemus, 2004). It is more likely that lower cortisol levels are related to attenuation in down-regulation of emotion circuitry while social stress was elevated. Another possibility is that during a task that required motivated performance of the participants, the lack of cortisol increase could be characterized by the “tend and befriend” response rather than the “fight or flight” response in female stress coping (Taylor et al., 2000). ANDR in a stressful context without social support decreased general arousal indicated by decreased cortisol level and performance level. ANDR might have attuned the females to the negative context they were situated in and disengaged themselves in performing the task and thereby the rise of cortisol level was prevented. This might interact with circulating sex hormones differently throughout menstrual cycle phases, which remains to be tested.

### Social Threat Anticipation and Amygdala

Regarding the neural correlates of anticipation and evaluation of social threat, we were particularly interested in amygdala activation. Activation/deactivation of the amygdala during psychosocial stress tasks has been reported previously (Pruessner et al., 2008; Bayer et al., 2014; Kogler et al., 2015). Amygdala activation was stronger during the pre-FB block, suggesting the processing of social evaluative threat was higher during the initial experience of social threat. Whereas stronger activation/less deactivation was detectable than during the later experience (post-FB block) across both groups. Recently, amygdala activation was associated with social threat detection (Bach et al., 2015), suggesting that stronger deactivation may indicate a more successful inhibition of emotional arousal during a cognitively challenging task.

The impact of social threat should be modulated by the degree of stress sensitivity. Previous neuroimaging studies addressing menstrual cycle effects on stress sensitivity in the amygdala observed stronger amygdala activation in the neutral compared to stress condition in ML-females (Ossewaarde et al., 2010). These findings suggest different underlying internal processing strategies during preliminary social threat assessment among females in different menstrual phases. Implicit exposure to ANDR may augment an initial sensitivity to social stress that promotes heightened awareness and vigilance, particularly in ML-females. Since there were no group differences in mood change, we speculated that this might be due to the interaction of ANDR and individual differences during anticipatory cognitive reappraisal acting as an emotional arousal buffer (Gaab et al., 2005). Elsewhere, self-esteem was suggested to be one of these central processes that buffers anticipation of social threat (Taylor et al., 2008). We found a significant negative correlation between subjective competence and amygdala activation in initial evaluation of the emerging social threat in ML-females. The implicit impact of ANDR on subjective competence ratings in ML-females converged with the decreased performance level. This may be attributable to the anticipation of a bad outcome in the MIST under the expectation and the pressure to perform as social threat processing was enhanced. Taken together, this complement the previously reported effect of increased stress sensitivity as a function of progesterone (Ossewaarde et al., 2010) as well as the increased inhibition of social threat processing by higher self-esteem (Taylor et al., 2008).

### Menstrual Cycle and ANDR Modulated Hippocampal Stress Reaction

Our data support the assumption that the neural stress reaction was modulated by menstrual cycle phase (Derntl et al., 2008b; Andreano and Cahill, 2010; Bayer et al., 2014; Albert et al., 2015), with stronger hippocampal activation in ML-females compared to EF-females during stress. The group difference in hippocampus activation was in line with findings of heightened encoding of social threat in emotional processing studies. For example, disgust expressions were judged as more intense in females with high progesterone level (Conway et al., 2007), and a negative bias to angry and disgust faces was also reported in females during the luteal phase (Derntl et al., 2008a). More importantly, heightened sensitivity to nearby threat in social stimuli was not only modulated by the heightened decoding of social stimuli but also by the negative social context in females during the luteal phase (Maner and Miller, 2014) or after progesterone administration (van Wingen et al., 2008).

Notably, under ANDR ML-females were attuned to the presence of social evaluative threat indicated by enhanced hippocampus activation when compared to EF-females as predicted. Given that the hippocampus is crucial to the neural stress reaction and relates to learned aversive responses and stressful memories (Dolcos et al., 2004), hippocampus activation during the task could be associated with social threat. Our results were in the consensus of heightened encoding of social cues related to social threat in ML-females reported in the neuroimaging data that directly compared EF- and ML-females (Andreano and Cahill, 2010; Bayer et al., 2014). In ML-females, the neural stress reaction under ANDR was significantly positively correlated with negative mood and feeling less competent, which was not apparent in EF-females. This fact suggests that ML-females processed the social chemosignal differently during the stressful situation.

Furthermore, hippocampus activation was suggested to facilitate anticipatory function (Gupta et al., 2010). Stronger left hippocampal activation during anticipation of social threat could be due to initial increased demand for the stress task. Therefore, the hippocampus appeared to be critically involved in processing social evaluative threat. The hippocampus is often activated in response to exposure of social odors, this could possibly enhance the associated emotional processing. As shown previously, increased emotional memory related activation of the hippocampus might be potentially related to increased progesterone levels (Andreano and Cahill, 2010). ANDR may act on the same neural regions that are dense with other gonadal hormone receptors. It is assumed that ANDR is synthesized from pregnenolone (Baum and Bakker, 2013), a precursor for a wide range of neurosteroids including progesterone (Soucy et al., 2003). It is possible that higher amount of available progesterone in the mid-luteal phase may potentially increase expression of ANDR within the hippocampus or other neural circuits critical for psychosocial stress. Thereby it may enhance encoding of sensory and emotional input. This hypothesis needs to be tested in future studies by directly addressing the effect of ANDR and changes in sex hormone levels on hippocampus activation in stressful contexts.

Mixed gender sample MIST studies proposed hippocampus activation as a crucial cortisol release inhibitor (Pruessner et al., 2008): the posterior hippocampus was activated while the anterior hippocampus was deactivated simultaneously in both cortisol responders and the whole sample (Dedovic et al., 2009). A recent MIST study with a female-only sample comparing ovulation and early follicular phase revealed that hippocampus activation was stronger in females with higher estradiol level, regardless of menstrual cycle phase. However, an association of cortisol reactivity to prefrontal cortex activation, but not hippocampus activation, was observed (Albert et al., 2015). In contrary, cortisol levels at T1 and T2 in our sample were high at both time points regardless of menstrual cycle phase, despite the increased posterior hippocampus activation in ML-females. These contrasting patterns suggest that high progesterone in ML-females and high estradiol in females during the mid-cycle may modulate hippocampus and its role in regulating HPA reactivity in a more complicated manner than it was suggested in the mixed sample data. Future studies should investigate female stress reactions across early follicular, mid-cycle and mid-luteal phases longitudinally to delineate the complexity of the role of the different female hormones on HPA-regulation at different subregions of the hippocampus.

### Other Considerations and Limitations

Odor application methods vary within the reported studies; however, we consider our patch application at the upper lip to be closer to spontaneous and naturalistic exposure (Sobel et al., 1999). The dosage for a specific emotional arousal effect and hormonal changes should be further substantiated. However, one cannot rule out the possibility that participants who did not smell ANDR and responded to social evaluation with a robust stress response may be characterized by the polymorphism of an olfactory receptor (OR7D4) that is selectively activated by the ANDR (Keller et al., 2007). Therefore, the genetic predisposition of the participants might have confounded the menstrual cycle effect on ANDR. Moreover, humans do not possess a functional vomeronasal organ (VNO), the typical processing site for chemosensory signals in rodents. Nevertheless, processing of chemosignals remained intact in rodents with VNO-ectomy (Pfeiffer and Johnston, 1994). In humans, specific receptors (TAAR) in the olfactory tissue are suspected to mediate subsequent behavior by integrating social information (Liberles and Buck, 2006). Hence, the significance of chemosignals in social threat communication could be modulated by multiple factors including differences in emotional information processing across menstrual cycle phases (see Lübke and Pause, 2015).

One major limitation of the study is that menstrual cycle was only verified by self-report, rather than salivary or serum hormone levels. Although we were careful to obtain self-reports at multiple times (recruitment, notification for appointment and on both days of testing as well as after testing), this remains sub-optimal. Data regarding actual ovarian hormone levels (progesterone/estradiol) would further validate the menstrual cycle effect as a specific function of progesterone at the neural level in ML-females. Another limitation is the age range of the sample, which would likely increase between-subject variability in hormone levels at each point in the menstrual cycle. These limitations also preclude us from explaining the lack of group difference in cortisol levels (under PLAC exposure) as previously reported (Kirschbaum et al., 1999). However, our findings are in line with more recent data from Duchesne and Pruessner (2013) with no female group difference in cortisol levels. Similarly, Albert et al. (2015) reported no influence of estradiol or progesterone on post-stress cortisol levels within their female groups. It is possible that individual differences in stress anticipation buffered the baseline cortisol level as we observed higher baseline cortisol levels with no differences between T1 and T2 in our study. Additional influences might be the basal levels of sex steroids (estradiol, progesterone and testosterone) that may act against the hippocampus negative feedback on the magnitude of cortisol release across different individuals. A recent study with a large sample has pointed out that the sex difference in cortisol reactivity was attenuated when gonadal hormone levels were adjusted (Juster et al., 2015b). Assessment of sex hormones across different age groups would be necessary to control for possible hormone-stress and hormone-hormone interactions. To reliably induce post-stress cortisol increase, face to face social threat should be incorporated into future MIST studies to maximize the impact of social threat. Regarding other confounds, our interview screenings did not show current mood or physical symptoms related to premenstrual dysphoric disorder, yet, survey of previous premenstrual syndrome is recommended. Another possible confound could be previous use of oral contraceptives, which we did not assess. As hypothesized, the individual differences that enhanced social threat processes appeared to be strongly correlated with neural stress in the a priori defined regions. However, our sample size could be too small to bear with the current design with some considerable inter-/intra-individual variances in the variables that we tested [e.g., while ML females showed a significantly decreased performance level under ANDR (*p* = 0.011), EF-females showed a similar reduction that did not reach significance (*p* = 0.098)]. Finally, we acknowledge that our MRI protocol was not sensitive enough to detect hypothalamus activation in all participants to test for the possible trigger of changes in cortisol levels. However, other comparable studies using a similar but higher threshold (916 μM/0.916 mM) also did not report hypothalamic activation in an fMRI olfactory study with 19 females tested at their periovulation (Zhou and Chen, 2008).

## Conclusion

The present study integrated a social chemosensory compound (ANDR) and a psychosocial stress task in the confined neuroimaging environment. We have demonstrated that stress anticipation and social threat sensitivity were enhanced by ANDR in ML-females, apparent in stronger amygdala activation associated with decreased subjective competence ratings, stronger hippocampal activation associated with negative mood, as well as lower performance levels. Taken together, the stress reaction and the effects of ANDR differed across the menstrual cycle and thus our results highlight the importance of integrating menstrual cycle phase in stress and chemosensory research.

## Author Contributions

KC collected the data, performed data analyses, and prepared the manuscript. FP helped with data collection and processed the skin conductance data. LK, SR, and BT helped with data interpretation and reviewed the manuscript. JF and BD designed the study and supervised data collection as well as preparation of the manuscript.

## Conflict of Interest Statement

The authors declare that the research was conducted in the absence of any commercial or financial relationships that could be construed as a potential conflict of interest.
