# Corrigendum: The Influence of Menstrual Cycle and Androstadienone on Female Stress Reactions: An fMRI Study

**DOI:** 10.3389/fnhum.2016.00293

**Published:** 2016-06-21

**Authors:** Ka Chun Chung, Felix Peisen, Lydia Kogler, Sina Radke, Bruce Turetsky, Jessica Freiherr, Birgit Derntl

**Affiliations:** ^1^Department of Psychiatry, Psychotherapy and Psychosomatics, Medical Faculty, RWTH Aachen UniversityAachen, Germany; ^2^Jülich Aachen Research Alliance – Translational Brain MedicineAachen, Germany; ^3^Department of Psychiatry and Psychotherapy, Medical Faculty, University of TübingenTübingen, Germany; ^4^Neuropsychiatry Division, Department of Psychiatry, University of PennsylvaniaPhiladelphia, PA, USA; ^5^Diagnostic and Interventional Neuroradiology, Medical Faculty, RWTH Aachen UniversityAachen, Germany; ^6^Fraunhofer Institute for Process Engineering and Packaging IVVFreising, Germany; ^7^Institute for Neuroscience and Medicine (INM-1), Research Center JuelichJuelich, Germany

**Keywords:** menstrual cycle, stress, social threat, cortisol, hippocampus, amygdala

Corrigendum on: Author Contributions

In the original article, the use of “prepared the manuscript” and “supervised data collection as well as preparation of the manuscript” in the Author Contributions Statement was unintentionally vague and potentially confusing. The authors apologize for the lack of clarity. This change does not influence the scientific conclusions of the article in any way. The full corrected Author Contributions Statement appears below.

KC collected the data, performed data analyses, and wrote the manuscript. FP helped with data collection and processed the skin conductance data. LK, SR, and BT helped with data interpretation and reviewed the manuscript. JF and BD designed the study and supervised data collection as well as reviewed the manuscript.

## Funding

The work in this manuscript was supported by the Medical Faculty, RWTH Aachen, Aachen, Germany (START 691302) and the Deutsche Forschungsgemeinschaft (DFG, IRTG 1328). BD was further supported by the Austrian Science Fund (FWF P23533).

### Conflict of interest statement

The authors declare that the research was conducted in the absence of any commercial or financial relationships that could be construed as a potential conflict of interest.

