# Supplementary material for: The Influence of Menstrual Cycle and Androstadienone on Female Stress Reactions: An fMRI Study
Source: Front Hum Neurosci. 2016 Feb 16;10:44. doi: 10.3389/fnhum.2016.00044 (PMC4754653; doi:10.3389/fnhum.2016.00044)
Supplement: Supplementary file 1 [file Table_1.DOCX]

Supplementary Material

**The influence of menstrual cycle and androstadienone on female stress reactions: an fMRI study.**

**Chung, K.C.^*^, Peisen, F., Kogler, L., Radke, S., Turetsky, B., Freiherr, J. & Derntl, B.**

*** Correspondence:** Ka Chun Chung, MSc: *e-mail:* kchung@ukaachen.de

# Menstrual cycle information of mid-luteal females

The details of testing day (day within the actual cycle) and days until the next menstruation as well as general length of menstruation for all mid-luteal women were listed in the Supplementary Table 1.

# Whole brain contrasts and contrasts masked by the anatomical ROIs

Regarding the whole brain analysis, we observed stronger stress>control activation of right middle temporal gyrus (MTG), superior frontal gyrus (SFG), precuneus, cerebellum, brainstem and left precentral gyrus and pre-motor area under ANDR exposure across the whole group (n=28). These regions are commonly reported in psychosocial stress studies with the fronto-parietal regions being associated with task relevant processing including sustained and switching attention, memory retrieval and motor coordination (Kogler et al., 2015b; Pruessner et al., 2008; Wang et al., 2007). Similar aforementioned activations were observed under PLAC exposure with a significant cluster at posterior cingulate gyrus. However, there were no treatment differences or other group-by-treatment interactions at the whole brain level, probably due to the low ANDR intensity and the challenging cognitive task. The details of activation coordinates, cluster size and statistics are added in the Supplementary Table 1 in the revised Supplementary Materials.

In the stress > control contrast (ANDR) masked by the anatomical ROIs for bilateral hippocampus, a significant cluster at right posterior hippocampus emerged (whole brain FWE correction at p=0.05). Again, the right hippocampus activation remained significant in the contrast ML > EF under ANDR exposure (across control and stress condition). This is congruent to the treatment-by-group interaction observed in the ROI analyses, indicating stronger hippocampal activation in ML-females compared to EF-females.

Contrasts including ROI masks of the bilateral amygdala did not reveal any significant clusters at the applied threshold.

The details of activation coordinates, cluster size and statistics were listed in the Supplementary Table 2.

# Threshold and discrimination test of ANDR

Six participants of each group were able to discriminate between ANDR at 250μM and PLAC in a three-repetition, forced-choice discrimination test (one ANDR, two PLAC solutions in a 20ml dilution presented with a 60ml glass jars). For the threshold test, we used the dilution-steps that are explained in great detail in Burke et al. (2012) and an extra step at 250 μM (= 0.25 mM). For sensitivity, a seven steps staircase test was conducted. Two consecutive correct identifications of a target in a triplet (one target, two control stimuli) were determined as the detection threshold. Surprisingly, most participants detected ANDR in a polypropylene dilution at the lowest dilution (see Supplementary Table 3). However, the Mann-Whitney test was applied as the data was not normally distributed and showed with no group difference between early follicular females (EF) and mid-luteal females (ML; U = 46, p = 0.066 two-tailed) occurred.

Regardless of menstrual cycle phases, our current sample showed higher sensitivity to the detection concentration of 250μM reported previously (Boulkroune et al., 2007; Lundström et al., 2003). Limited by our lower sample size of participants with lower sensitivity to ANDR in the repeated measure design, we were not able to compare participants with high vs. low sensitivity to ANDR. To gain a better understanding, future studies should aim to measure participants in highly and lowly sensitive groups. One possibility is that the human olfactory receptor, OR7D4, a canonical olfactory G protein-coupled receptor, could affect pleasantness and intensity of ANDR perception (Keller et al., 2007).

# Skin conductance response (SCR)

Two 8 mm (in diameter) AG/AGCl electrodes housed in plastic cup were filled with isotonic electrode paste (0.5% saline in a neutral base) (Med Associates TD-246) and were placed at the palmar side and middle phalanges of the index and middle finger of the non-dominant (left) hand. SCR data were acquired at a sampling rate of 5000 Hz in DC mode using a bipolar BrainAmp ExG MR amplifier and BrainVision Recorder (Brain Products, Gilching, Germany). Offline data analyses included downsampling procedure to 2 Hz, artifact reduction using spline interpolation, and extraction of phasic components from tonic activity based on continuous decomposition analysis (Benedek and Kaernbach, 2010) implemented in Ledalab© software (Leipzig, Germany). Phasic SCR were defined as deflections above 0.02 mS and were analyzed with respect to the parameter “nSCR” (number of SCRs) within a post-stimulus epoch of 1–72 sec (Boucsein et al., 2012; Kogler et al., 2015a). Normalized SCR data was analyzed by a 2 x 2 x 2 rmANOVA with the factors treatment (ANDR/PLAC), condition (control/stress) and group (EF/ML).

Due to hardware problems during data collection, phasic SCR responses of only 14 females (EF, n=6; ML, n=8) were analyzed. A significant main effect of condition (F_1;12_ = 11.14, p = 0.006, $\eta_{p}^{2}$ = 0.481) emerged, demonstrating higher nSCR responses in the stress compared to the control condition. However, significant group and treatment effects were not established (all F_1;12_ ≤ 0.484, ps ≥ 0.500).

# References

Benedek, M., and Kaernbach, C. (2010). A continuous measure of phasic electrodermal activity. *J Neurosci Methods* 190, 80–91. doi:10.1016/j.jneumeth.2010.04.028.

Boucsein, W., Fowles, D. C., Grimnes, S., Ben-Shakhar, G., roth, W. T., Dawson, M. E., et al. (2012). Publication recommendations for electrodermal measurements. *Lit. Discuss* 49, 1017–1034. doi:10.1111/j.1469-8986.2012.01384.x.

Boulkroune, N. N., Wang, L. L., March, A. A., Walker, N. N., and Jacob, T. J. C. T. (2007). Repetitive olfactory exposure to the biologically significant steroid androstadienone causes a hedonic shift and gender dimorphic changes in olfactory-evoked potentials. *Neuropsychopharmacology* 32, 1822–1829. doi:10.1038/sj.npp.1301303.

Keller, A., Zhuang, H., Chi, Q., Vosshall, L. B., and Matsunami, H. (2007). Genetic variation in a human odorant receptor alters odour perception. *Nature* 449, 468–472. doi:10.1038/nature06162.

Kogler, L., Gur, R. C., and Derntl, B. (2015a). Sex differences in cognitive regulation of psychosocial achievement stress: Brain and behavior. *Hum. Brain Mapp.* 36, 1028–1042. doi:10.1002/hbm.22683.

Kogler, L., Mueller, V. I., Chang, A., Eickhoff, S. B., Fox, P. T., Gur, R. C., et al. (2015b). Psychosocial versus physiological stress - Meta-analyses on deactivations and activations of the neural correlates of stress reactions. *NeuroImage* 119, 235–251. doi:10.1016/j.neuroimage.2015.06.059.

Lundström, J. N., Hummel, T., and Olsson, M. J. (2003). Individual Differences in Sensitivity to the Odor of 4,16-Androstadien-3-one. *Chem Senses* 28, 643–650. doi:10.1093/chemse/bjg057.

Pruessner, J. C., Dedovic, K., Khalili-Mahani, N., Engert, V., Pruessner, M., Buss, C., et al. (2008). Deactivation of the Limbic System During Acute Psychosocial Stress: Evidence from Positron Emission Tomography and Functional Magnetic Resonance Imaging Studies. *Biol. Psychiatry* 63, 234–240. doi:10.1016/j.biopsych.2007.04.041.

Wang, J., Korczykowski, M., Rao, H., Fan, Y., Pluta, J., Gur, R. C., et al. (2007). Gender difference in neural response to psychological stress. *Soc. Cogn. Affect. Neurosci.* 2, 227–239. doi:10.1093/scan/nsm018.

Supplementary Table 1. Table indicating usual cycle length, testing day (ANDR or PLAC) and days until the next menstruation following the testing session for each mid-luteal woman. This information was confirmed via telephone calls or email contact.

| ML-women | Usual cycle length | Testing day within the menstrual cycle (PLAC) | Testing day until the next menstruation (PLAC) | Testing day within the menstrual cycle (ANDR) | Testing day until the next menstruation (ANDR) |
| --- | --- | --- | --- | --- | --- |
| 1 | 26 | 21 | 5 | 24 | 3 |
| 2 | 26 | 21 | 5 | 22 | 4 |
| 3 | 28 | 22 | 5 | 23 | 5 |
| 4 | 28 | 24 | 4 | 20 | 8 |
| 5 | 28 | 23 | 5 | 20 | 8 |
| 6 | 28 | 22 | 6 | 22 | 6 |
| 7 | 29 | 23 | 7 | 20 | 9 |
| 8 | 29 | 23 | 6 | 20 | 9 |
| 9 | 29 | 24 | 4 | 21 | 8 |
| 10 | 29 | 21 | 8 | 20 | 9 |
| 11 | 30 | 25 | 4 | 25 | 6 |
| 12 | 30 | 24 | 6 | 25 | 5 |
| 13 | 30 | 21 | 9 | 25 | 5 |
| 14 | 30 | 20 | 10 | 23 | 7 |
| 15 | 30 | 25 | 4 | 24 | 6 |
| 16 | 30 | 22 | 8 | 23 | 6 |

**Supplementary Table 2.** Whole brain contrasts showing stronger activation in the stress compared to control condition for the whole sample under androstadienone (ANDR) and placebo (PLAC) exposure (n=28); stronger activation in the stress>control contrast (masked by anatomical mask of bilateral hippocampus [Hipp.]) under androstadienone (ANDR); stronger activations (across control and stress conditions) in mid-luteal females (ML; n=15) compared to early follicular females (EF; n=13) under ANDR. No significant clusters emerged in contrasts masked by anatomical mask of bilateral amygdala.

|  | **MNI** | | | **k** | **Z** | **Sig.** | **Region** |
| --- | --- | --- | --- | --- | --- | --- | --- |
| **Contrasts** | **X** | **Y** | **Z** |  |  |  |  |
| **Whole sample** |  |  |  |  |  |  |  |
| **ANDR** |  |  |  |  |  |  |  |
| Stress > control | 51 | -66 | -1 | 230 | 7.02 | <0.001 | R. MTG |
|  | 24 | -3 | 55 | 55 | 5.32 | 0.001 | R. SFG |
|  | 18 | -63 | 55 | 39 | 5.05 | 0.003 | R. precuneus |
|  | 34 | -39 | -11 | 7 | 4.94 | 0.005 | R. parahippocampal gyrus |
|  | 34 | -46 | -24 | 29 | 4.82 | 0.009 | R. cerebellum 6 |
|  | 21 | 33 | -12 | 10 | 4.77 | 0.010 | R. OFC |
|  | 8 | -26 | -7 | 7 | 4.77 | 0.010 | R. brainstem/PAG |
|  | -35 | -6 | 49 | 7 | 4.76 | 0.011 | L. precentral gyrus |
|  | 34 | -79 | 19 | 22 | 4.69 | 0.015 | R. MOG |
|  | 8 | -66 | -14 | 9 | 4.62 | 0.019 | R. cerebellum 6 |
|  | -42 | -66 | 6 | 7 | 4.59 | 0.022 | L. MOG |
|  | -19 | -6 | 62 | 3 | 4.55 | 0.026 | L. pre-motor |
|  | 37 | -6 | 45 | 4 | 4.54 | 0.028 | L. pre-motor |
|  |  |  |  |  |  |  |  |
| Control > stress | No sig. activation | | | | | | |
|  |  | | | | | | |
| **PLAC** |  |  |  |  |  |  |  |
| Stress > control | 44 | -59 | 9 | 816 | 6.68 | <0.001 | R. MTG |
|  | 24 | 0 | 52 | 328 | 6.18 | <0.001 | R. precentral gyrus |
|  | 54 | -39 | 29 | 93 | 5.59 | <0.001 | R. STG |
|  | -28 | -53 | -14 | 166 | 5.49 | <0.001 | R. fusiform gyrus |
|  | -42 | -10 | 49 | 87 | 5.49 | <0.001 | L. precentral gyrus |
|  | -45 | -72 | 12 | 150 | 5.34 | 0.001 | L. MOG |
|  | 8 | -39 | 45 | 123 | 5.20 | 0.001 | R. precuneus |
|  | 4 | -63 | -30 | 113 | 5.04 | 0.003 | R. cerebellum 6 |
|  | 8 | -26 | -7 | 14 | 4.94 | 0.005 | R. brainstem/PAG |
|  | 18 | 43 | -1 | 10 | 4.77 | 0.011 | R. OFC |
|  | 37 | 33 | -1 | 8 | 4.71 | 0.013 | R. IFG (pars triangularis) |
|  | -12 | -20 | 39 | 5 | 4.58 | 0.023 | L. cingulate gyrus (posterior) |
|  | -12 | 7 | 42 | 4 | 4.54 | 0.027 | L. pre-motor |
|  |  | | | | | | |
| Control > stress | No sig. activation | | | | | | |
|  | | | | | | | |
| **Contrasts masked by anatomical defined hippocampus** | | | | | | | |
| **Whole sample** |  |  |  |  |  |  |  |
| **ANDR** |  |  |  |  |  |  |  |
| Stress > control | 34 | -33 | -11 | 27 | 5.27 | 0.001 | R. post. Hipp. |
| Control > stress | No sig. activation | | | | | | |
| **PLAC** |  |  |  |  |  |  |  |
| Stress > control | No sig. activation | | | | | | |
| Control > stress | No sig. activation | | | | | | |
| **ANDR** |  |  |  |  |  |  |  |
| ML > EF | 21 | -30 | -6 | 3 | 4.72 | 0.013 | R. post. Hipp. |
| EF > ML | No sig. activation | | | | | | |
| **PLAC** |  |  |  |  |  |  |  |
| ML > EF | No sig. activation | | | | | | |
| EF > ML | No sig. activation | | | | | | |
|  |  |  |  |  |  |  |  |
| **Contrasts masked by anatomical defined amygdala** | | | | | | | |
|  | No sig. activation | | | | | | |
|  | | | | | | | |
|  |  |  |  |  |  |  |  |

Note: Middle temporal gyrus (MTG), superior frontal gyrus (SFG), periaqueductal gray (PAG), orbitofrontal cortex (OFC), middle occipital gyrus (MOG), superior temporal gyrus (STG), inferior frontal gyrus (IFG), pre-motor area (pre-motor), anterior cingulate cortex (ACC), hippocampus (Hipp.). Results of the whole-brain analyses were thresholded at p < .05 family-wise error (FWE) corrected.

**Supplementary Table 3.** Threshold (mM) of androstadienone (ANDR) detected by both in early follicular females (EF, n=15) and mid-luteal females (ML, n=15). One participant did not complete the test and four participants were anosmic to ANDR.

| (mM) | EF | ML |
| --- | --- | --- |
| 0.0001 | 8 | 4 |
| 0.001 | 3 | 1 |
| 0.01 | 1 | 4 |
| 0.1 | 0 | 1 |
| 0.25 | 0 | 1 |
| 1 | 1 | 2 |
| 10 | 0 | 0 |
| anosmic | 2 | 2 |
